# The Call for Reconstruction

**Published:** 1919-01-04

**Authors:** 


### THE MATRONS' AND SISTERS' DEPARTMENT.

### THE CALL FOR RECONSTRUCTION.

THE New Year dawns amid quite a remarkable wave of hope and determination. Dissatisfaction with things as they are has led to a very eager awakening to the possibilities which lie in the future. It is more than probable that much of the legislation anticipated by reformers will be of a permissive character. It is not the English way to send State-appointed officials to do for the country things which local men and women are too ignorant or lazy to do for themselves. Local bodies are to be stimulated by grants in aid, but the brains and propulsive power to use them must be home-grown. This principle it is which has lent the English-speaking peoples their tremendous force. It is a principle which extends to every branch of work and is seen in active operation in all our institutions for the sick. It is a principle which has inspired the organisation of the nursing profession from the time when it first began to feel its wings growing. Unless we are to lose a great inspiring force in the progress of nursing this democratic principle must be kept intact after the battle for State recognition has been won.

Briefly, the effect of the English principle that it is better men should plan things for themselves, instead of having orders from headquarters, is to set the minds of the workers actively working. If all in an establishment, even the heads, are working as part of a machine under laws which can never be modified or altered, there settles upon everyone a mould of acquiescence in remediable evils which saps all vitality. Many clear-sighted matrons and sisters have experienced this damping and most exasperating influence in the course of their Army service. It is well called red tape, for it effectually ties up the faculties and fits them to be laid on the shelf.

The call to reconstruction is to untie the strings of the mind. It is a call, not to restless discontent, but to silent steadfast inquiry into existing conditions, their defects and their possible alteration. There is no institution in the country, we say it deliberately, which does not contain remediable defects. Almost any manager would say if confronted by them, "Oh, if we had the money," and would leave it at that. But money is not the first requisite; it is brains. It has been our lot to watch the progress of many wonderful changes in the hospitals of Great Britain during the last thirty or forty years. In scarcely any instance have they been due to the initiative lent by money. Occasionally it may have happened that a large donation or legacy may have set managers to work to effect alterations. But as a rule the schemes which have revolutionised hospital treatment and the nursing of the sick have originated in the minds of the workers themselves, have spread imperceptibly when once planted in receptive soil, and have come to accomplishment, none can say how, by virtue of some innate vital principle in ideas which may not be thwarted by circumstances.

The call of the future is for ideas. All over the country are women placed in authority, some over large and some over very small groups of workers. Are they satisfied with the conditions under which they and their subordinates are working? If not, why not? What is wrong with the system or with the building? To get a clear impression of what irks each individual at any particular moment in the day is to have gained a strong vantage-point for reform. When this has been reached, when the one in authority knows exactly where each of her subordinates finds her energy sapped, then comes the survey of the whole situation, which perhaps after months of secret cogitation is not complete. To think out a reform even of the simplest character is the most difficult exercise with which the mind can grapple. But in this lies the secret of reconstruction in nursing as in all other matters. To frame a good plan is to frame the lever which moves mountains.

